# Comparison of clinical results between transpulmonary thermodilution monitoring and conventional methods in cardiac surgery: An observational study

**DOI:** 10.1097/MD.0000000000040884

**Published:** 2024-12-20

**Authors:** Serkan Ertugay, Ümit Kahraman, Emrah Oğuz, Emre Demir, Esin Öztürk, Nüzhet Seden Kocabaş, Osman Nuri Tuncer, Pelin Öztürk, Mustafa Özbaran

**Affiliations:** a Department of Cardiovascular Surgery, Ege University Faculty of Medicine, İzmir, Turkey; b Department of Cardiology, Ege University Faculty of Medicine, İzmir, Turkey; c Department of Anesthesiology and Reanimation, Ege University Faculty of Medicine, İzmir, Turkey.

**Keywords:** cardiac surgery, goal-directed therapy, hemodynamic management, pulse contour analysis, thermodilution

## Abstract

The Pulse Index Contour Continuous Cardiac Output (PICCO) module provides advanced and continuous monitoring of cardiac output through the use of arterial pulse contour analysis and transpulmonary thermodilution. The objective of this study was to compare the early postoperative outcomes of patients who were monitored using the conventional method and the pulse contour analysis method. A prospective observational study was conducted involving 45 patients who underwent cardiac surgery between 2020 and 2022. Patients were randomly assigned to either Group P (PICCO) or Group C (conventional). In the PICCO cohort, a femoral artery cannula was inserted for the continuous recording and management of hemodynamic data, in accordance with the decision-making algorithm of the module. In the conventional group, cannulation of the radial artery and jugular vein was performed. The postoperative hemodynamic and clinical data are subjected to analysis. The utilization of dopamine was markedly diminished in Group P at both the 0- and 6-hour postoperative intervals, whereas the administration of dobutamine was observed to be elevated (*P* = .008). The frequency of red packed cell transfusions was higher in Group C at postoperative hour 0. Hemodynamic data indicated a 42% increase in cardiac index and a 33% decrease in systemic vascular resistance, along with a 33% increase in global ejection fraction in patients monitored with PICCO. The mortality rates observed in the 2 groups were not statistically different. The implementation of advanced monitoring techniques, specifically the PICCO module, led to notable enhancements in hemodynamic parameters. The utilization of this technique may prove advantageous in guiding inotrope selection and transfusion decisions during the initial postoperative period. However, it is important to note that morbidity and mortality rates remain comparable.

## 1. Introduction

Arterial and central venous pressure monitoring are commonly employed in the management of hemodynamic fluctuations, volume imbalances, and myocardial dysfunction during the perioperative period of cardiac surgery. Pulmonary artery catheters represent the gold standard for advanced monitoring. However, measuring the left atrial pressure by placing the catheter tip distal to the pulmonary artery is an invasive procedure that carries the risk of complications, including arrhythmias, bacteremia, thrombus formation, pulmonary infarction, pulmonary artery rupture, bleeding, catheter tangling, and pulmonary valve damage.^[1]^ The necessity for repetitive measurements arises from the inherent limitations of continuous monitoring. It is therefore imperative that future research focus on the development of less invasive, continuous, and accurate measurement technologies.

One such technology is continuous monitoring of cardiac output through transpulmonary thermodilution and pulse contour analysis (PICCO, PULSION Medical Systems SE in Feldkirchen, Germany). The Pulse Index Contour Continuous Cardiac Output (PICCO) system employs the Stewart–Hamilton principle to measure cardiac output, a methodology distinct from that utilized by the Swan-Ganz system. A cold saline solution is injected into the jugular vein, and a thermistor is used to detect the decrease in blood temperature in the systemic artery. The system is capable of continuously measuring a number of important parameters, including cardiac output, extravascular lung water (EVLW), global end-diastolic volume, global end-diastolic volume index (GEDVI), intrathoracic blood volume, Cardiac Function Index, and global ejection fraction (GEF).^[2]^ This device is widely accepted as a physiological monitor due to its decision-making algorithm for fluid and vasopressor management. Nevertheless, the clinical advantages of these monitoring systems in cardiac surgery remain uncertain.

The objective of this study was to compare the early postoperative clinical outcomes of patients who were monitored using the conventional and transpulmonary thermodilution pulse contour analysis methods.

## 2. Materials and methods

This is a prospective, observational study conducted at the Department of Cardiovascular Surgery of Ege University between 2019 and 2020. The study was approved by the Ege University Medical Research Ethics Committee (document number 18-12.1T/9).

The study population consisted of patients who provided written informed consent and underwent cardiac surgery performed by a single surgeon in the Cardiovascular Surgery Department. The study initially included 25 patients in each group; however, due to technical failure of the PICCO catheter, 5 patients were excluded. The final sample consisted of 45 patients, who were divided into 2 groups. The patients were divided into 2 groups: Group P, which included 20 patients who underwent PICCO insertion, and Group C, which included 25 patients who underwent conventional insertion.

### 2.1. Study protocol

Both groups underwent hemodynamic monitoring by inserting a central venous catheter and an arterial catheter. Patients in Group C had arterial pressure (radial artery) and central venous pressure (right internal jugular vein), and hemodynamic management was provided using conventional methods. In Group P, an additional 5 F (French) femoral artery cannula was inserted. Upon connection to the PICCO monitor, the patients’ biometric data, estimated body weight, height, sex, and injected volume were entered into the system. The venous injection port was connected to a jugular venous catheter. Measurements were taken while the patient was in the supine position, with the pressure transducer zeroed against atmospheric pressure at the level of the right heart. Mechanical ventilation was applied in pressure support mode. For calibration purposes, a thermodilution curve was created by injecting 15 mL of 0.9% NaCl at a temperature below 8°C through the distal lumen of the jugular venous catheter. The injections were administered thrice, and continuous measurements were performed. For patients with PICCO, measurements were taken before and after cardiopulmonary bypass, at the 6th hour post-operation, and after extubation. Group P followed an algorithmic approach using the PICCO module for hemodynamic management.

Demographic information, comorbidities, laboratory analyses, and operative and postoperative characteristics including PICCO parameters were recorded. The main objective of this study was to evaluate the use of inotropic agents and red-packed cells as primary endpoints. The secondary endpoints included duration of mechanical ventilator support, complications, length of intensive care and hospital stay, and mortality.

### 2.2. Statistical analysis

The preoperative characteristics of the patients were summarized as mean and standard deviation for numerical variables and as frequency and percentage values for categorical variables. In this study, the odds ratio and 95% probability confidence interval were used as risk criteria. The Mann–Whitney *U* test was used to compare medians (or means) between Groups P and C based on relevant variables. The change percentages were calculated to examine the changes in relevant variables depending on the operation time. Chi-square analyses were used to compare preoperative and postoperative values between the groups.

## 3. Results

Group P consisted of 20 patients, with a mean age of 58 ± 11 years and a female prevalence of 55%. Group C included 25 patients, with a mean age of 57 ± 11 years and a female prevalence of 65%. No significant differences were observed in age or sex between the 2 groups. However, a higher incidence of hypertension, chronic obstructive pulmonary disease (COPD), and diabetes mellitus (DM) was observed in Group P. Furthermore, the preoperative left ventricular ejection fraction was marginally lower in Group P (44.1% vs 50%, *P* = .003). A comparison of the remaining preoperative characteristics is presented in Table 1.

**Table 1:** Preoperative patient characteristics.

	Group P (PİCCO) n = 20 pts	Group C (conventional) n = 25 pts	*P* value	Odds ratio	%95 confidence interval
Age (yr)	58 ± 1	57 ± 1	.46		
Gender (woman)	11 (55%)	16 (65%)	.55	1.45	0.43–4.8
Body surface area	1.8 ± 0.1	1.8 ± 0.1	.85		
Diabetes	8 (40%)	3 (12%)	**.03**	0.205	0.04–0.918
Hypertension	16 (80%)	11 (44%)	**.015**	0.196	0.05–0.75
Hyperlipidemia	5 (25%)	2 (8%)	.12	0.261	0.04–1.5
Carotid artery stenosis	1 (5%)	–	.26	0.95	0.85–1.0
Chronic renal failure	3 (15%)	4 (16%)	.93	1.08	0.212–5.5
Liver disease	2 (10%)	–	.11	0.9	0.78–1
COPD	7 (35%)	1 (4%)	**.008**	0.77	0.009–0.7
Pre-op hemoglobin	12.4 ± 2.3	12.5 ± 1.6	.803		
Pre-op creatine	1.12 ± 0.7	0.96 ± 0.8	.166		
Pre-op albumin	41.9 ± 5.9	43.6 ± 4.07	.26		
Pre-op left ventricle ejection fraction	44 ± 10	53 ± 5	**.003**		
Operation type					
1: isolated valve	11 (55%)	18 (72%)			
2: CABG + valve	3 (15%)	1 (4%)			
3: multiple valve	6 (30%)	6 (24%)			

Bold values indicate statistically significant results.

CABG = coronary artery bypass grafting, COPD = chronic obstructive pulmonary disease, PICCO = Pulse Index Contour Continuous Cardiac Output, pre-op = preoperative.

Upon analysis of the intraoperative data, it was found that Group P exhibited significantly lower usage of dopamine at both 0 and 6 hours postoperatively, while the usage of dobutamine was higher. Moreover, the transfusion of red blood cells was more prevalent in Group C at postoperative hour 0. The data from both groups exhibited comparable statistical characteristics with regard to postoperative complications. Two patients in Group P died, whereas none in Group C did, a difference that was not statistically significant (*P* = .2). The postoperative findings are presented in Table 2.

**Table 2:** Intraoperative patient characteristics and postoperative complications.

	Group P (PICCO)	Group C (conventional)	*P* value	Odds ratio	%95 confidence interval
Number of patients	n = 20	n = 25			
PO 0th hour dopamine (5 mcg/kg and above)	8 (42%)	22 (91%)	**<.000**	15.125	2.73–83.6
PO 0th hour dobutamine (5 mcg/kg and above)	11 (55%)	4 (17%)	.008	0.164	0.041–0.66
PO 0th hour Adrenaline (0.1 mcg/kg and above)	5 (25%)	3 (13%)	.436	0.43	0.09–2
PO 0th hour noradrenaline (0.1 mcg/kg and above)	3 (15%)	6 (25%)	.477	1.89	0.4–8.8
PO 0th-hour glycerol trinitrate (1 mg/kg and above)	8 (40%)	5 (21%)	.165	0.39	0.1–1.5
PO 0th hour RPC transfusion (unit)	0	10 (35%)	**.004**	1.5	1.2–2.05
PO 6th hour dopamine (5 mcg/kg and above)	10 (53%)	14 (58%)	.708	1.3	0.3–4.2
PO 6th hour dobutamine (5 mcg/kg and above)	9 (47%)	3 (13%)	**.011**	0.16	0.03–0.72
PO 6th hour adrenaline (0.1 mcg/kg and above)	4 (21%)	2 (8%)	.38	0.34	0.05–2.1
PO 6th hour noradrenaline (0.1 mcg/kg and above)	2 (11%)	4 (17%)	.68	1.7	0.27–10.5
PO 6th hour glycerol trinitrate (1 mg/kg and above)	8 (42%)	9 (38%)	.76	0.83	0.2–2.8
PO 6th hour RBC transfusion (unit)	3 (16%)	9 (36%)	.14	3	0.68–13.16
Dopamine after extubation (5 mcg/kg and above)	10 (53%)	9 (36%)	.27	0.51	0.15–1.7
Dobutamine after extubation (5 mcg/kg and above)	8 (42%)	3 (13%)	**.035**	0.2	0.043–0.9
Adrenaline after extubation (0.1 mcg/kg and above)	4 (21%)	3 (13%)	.68	0.53	0.1–2.8
Noradrenaline after extubation (0.1 mcg/kg and above)	4 (21%)	5 (21%)	.99	0.99	0.2–4.4
Glycerol trinitrate after extubation (1 mg/kg and above)	7 (37%)	9 (38%)	.954	1029	0.29–3.6
RBC after extubation transfusion (unit)	4 (20%)	12 (48%)	.51	3.7	0.96–14.2
CPB duration (min)	128	149	.15		
Aortic cross clamp time (min)	100	106	.28		
Postoperative leukocyte	10.3	12.2	.33		
Postop hemoglobin	9.1	8.8	.32		
Newly developing arrhythmia	9 (47%)	12 (48%)	.97	1.03	0.31–3.4
Non-dialysis kidney failure	3 (15%)	2 (8%)	.65	0.5	0.07–3.3
Dialysis requiring kidney insufficiency	2 (10%)	1 (4%)	.6	0.37	0.03–4.5
Prolonged mechanical ventilation (>24 h)	6 (30%)	2 (8%)	.5	0.2	0.03–1.01
Non-invasive mechanical ventilation support	4 (20%)	2 (8%)	.2	0.3	0.05–2.13
Reintubation	5 (25%)	2 (8%)	.21	0.26	0.04–1.5
Newly developing liver disorder	2 (10%)	1 (4%)	.58	0.38	0.03–4.7
Pulmonary edema	3 (15%)	1 (4%)	.3	0.2	0.02–2.5
Re-exploration due to bleeding	3 (15%)	1 (4%)	.3	0.2	0.02–2.19
Infection	4 (20%)	1 (4%)	.15	0.17	0.02–1.6
Pneumonia	3 (15%)	1 (4%)	.3	0.2	0.02–2.47
Low cardiac output	5 (25%)	1 (4%)	.74	0.12	0.02–1.18
Need for IABP	1 (5%)	1 (4%)	.999	0.8	0.04–13.5
Sepsis	1 (5%)	1 (4%)	.999	0.8	0.04–13.5
In-hospital mortality	2 (10%)	1 (4%)	.58	0.38	0.03–4.47
30-d mortality	2 (10%)	1 (4%)	.19	0.9	0.78–1.04

Bold values indicate statistically significant results.

CPB = cardiopulmonary bypass, ERT = erythrocyte suspension, IABP = intra-aortic balloon pump, PICCO = Pulse Index Contour Continuous Cardiac Output, PO = postoperative, RBC = red blood cells.

A comparison of the PICCO data from patients in Group P revealed a 42.47% increase in the average CI, from 2.19 ± 1.02 L during the preoperative period to 3.12 ± 0.7 L after extubation. Additional alterations included a 33.9% reduction in the SVRI, a 33.9% increase in the mean GEF, a 35.2% decline in the average EVLW, and a 3.7% reduction in GEDVI (Table 3).

**Table 3:** The changes in the measured values of patients in Group P during and after the operation, and after extubation.

	Pre-op	Time of exit from CPB	Post-op 0th hour	Post-op 6 h	After extubation	Between Pre-op and post-extubation value change (%)
Average CI	2.19 ± 1.02	2.88 ± 0.7	2.6 ± 0.72	2.9 ± 0.58	3.12 ± 0.7	**42.47**
Average SVRI	3193 ± 1660	2303 ± 1377	2708 ± 1335	2242 ± 689	2109 ± 688	**−33.95**
Average GEF	14.58 ± 4.8	18.05 ± 6.35	17.63 ± 5.8	18.9 ± 5.3	19.53 ± 6.4	**33.96**
Average ELWI	13.44 ± 7.14	10.17 ± 2.7	9.5 ± 2.6	8.63 ± 1.7	8.7 ± 1.4	**−35.27**
Average GEDVI	742 ± 194	675 ± 153	682 ± 161	663 ± 118	714.6 ± 160.7	**−3.78**

Bold values indicate statistically significant results.

CI = confidence interval, CPB = cardiopulmonary bypass, EVLW = extravascular lung water, GEDVI = global end-diastolic volume index, GEF = global ejection fraction, SVRI = systemic vascular resistance index.

## 4. Discussion

The findings of this study indicate that dobutamine is the preferred inotropic agent in patients monitored by PICCO, as determined by the decision-making model. Furthermore, these patients exhibited a reduced requirement for red blood cell transfusions at the early stage of intensive care unit (ICU) admission, which may indicate the efficacy of goal-directed fluid management in Group P. With regard to clinical outcomes, both groups demonstrated comparable results, although Group P exhibited a higher prevalence of risk factors and a lower preoperative left ventricle ejection fraction.

While the perioperative mortality rate has decreased over time, the incidence of major cardiovascular complications remains a significant concern. It is of great importance to monitor hemodynamics in order to improve clinical outcomes, particularly in cases of low cardiac output.^[3,4]^ For advanced monitoring, a variety of techniques are employed, including invasive methods such as the Fick method, thermodilution, radioisotope analysis, contrast, and radionuclide angiography, as well as noninvasive alternatives such as pulse counter analysis, Doppler methods, transthoracic electrical bioimpedance measurement, ballistocardiography, and echocardiography.^[5]^ The selection of an appropriate method is dependent upon a number of factors, including the patient’s condition, hemodynamic parameters, the availability of clinical resources, and the preferences of the clinician. Although invasive methods are preferred for their ability to provide real-time and reliable cardiac output measurements, noninvasive methods offer several advantages, including a less invasive nature, ease of use, and the ability to measure a multitude of parameters.

A comprehensive, multicenter survey in Germany revealed that the most prevalent monitoring techniques employed in high-risk cardiac surgery patients included arterial and central venous catheterization, as well as transesophageal echocardiography.^[6]^ Despite the preference for pulmonary artery catheters in patients at high risk of cardiac surgery, national database analysis revealed no reduction in morbidity and mortality in this cohort.^[7]^ Furthermore, challenges associated with mechanical, thrombotic, and infectious complications, as well as the inability to accurately measure in cases of tricuspid valve insufficiency, have contributed to the adoption of less invasive methods.^[1]^

The Enhanced Recovery After Surgery (ERAS) guideline recommends the use of goal-directed fluid therapy (GDFT) guided by hemodynamic monitoring.^[8]^ A study by Kapoor et al^[9]^ examined the clinical efficacy of GDFT, noting a reduction in ventilation duration, length of stay in the ICU and hospital, and a significant decline in biomarkers when GDFT was applied. Another study demonstrated that postoperative goal-directed fluid therapy (GDFT) in patients following cardiac surgery was associated with a reduction in the incidence of acute kidney injury and a reduction in the duration of intensive care unit (ICU) and hospital stay.^[10]^ Finally, a meta-analysis conducted by Aya et al^[11]^ demonstrated a notable reduction in complications among patients undergoing cardiac surgery who received GDFT.

PICCO system parameters, including global end-diastolic volume and EVLW, are of paramount importance in determining the necessity of fluid therapy.^[12]^ A targeted treatment approach for hemodynamics allows for the optimal use of intravenous fluids and vasoactive support, thereby optimizing cardiac output and improving postoperative survival.^[13,14]^ An increased EVLW and pulmonary vascular permeability index (PVPI) are associated with edema and are known predictors of mortality.^[15]^ In our study, we observed significant changes in EVLW as a result of measurements made on CPB output, after extubation, and in the postoperative period in the PICCO group. These changes can be attributed to the algorithm for appropriate volume management and the necessary inotropic support as part of the targeted treatment. Furthermore, a reduced number of transfusions were administered during the initial postoperative hour in patients who underwent PICCO. The observed changes in PICCO parameters in our cohort included a 42% increase in CI, a 33.9% decrease in SVRI, a 33.9% increase in mean GEF, a 35% decrease in average EVLW, and a 3.7% decrease in GEDVI. These findings were similarly reported in the study by Lenkin et al,^[16]^ which was conducted in patients who underwent complex valve surgery.

Inotropic and vasopressor therapy, which is employed to augment cardiac function in the context of cardiovascular surgery, has been identified as the fundamental tenet of hemodynamic therapy. Nevertheless, evidence indicates that it increases myocardial oxygen consumption, causes arrhythmias, and disrupts the microcirculation.^[9,10,17]^ Nevertheless, its utilization is inevitable in instances of left ventricular dysfunction and low cardiac output. A survey from Germany indicated that dobutamine is the most preferred inotropic agent in cases of low cardiac output.^[6]^ Similarly, our study demonstrated that dobutamine usage for targeted inotropic purposes was higher in patients managed with the PICCO module, while dopamine usage was higher in patients managed with the conventional monitoring method during cardiac surgery. In a randomized control study reported by Smetkin et al,^[18]^ the use of PICCO allowed for early recognition of hypovolemia and myocardial depression during off-pump coronary bypass.

Given the limitations of this study, the relatively small sample size in both groups may have resulted in a low number of encountered events and no statistically significant differences between the groups. Furthermore, the analysis may have been influenced by preoperative risk factors such as chronic obstructive pulmonary disease (COPD) and diabetes mellitus (DM), as well as differences in left ventricular ejection fraction between the groups.

## 5. Conclusion

The implementation of advanced and continuous hemodynamic monitoring during cardiac surgery enables the acquisition of detailed data, which can then be utilized in a goal-directed management approach through the use of a decision model. While these data may prove beneficial for inotrope management and transfusion practices in the early postoperative period, they did not affect cardiac surgical complications or mortality.

## Author contributions

**Conceptualization:** Serkan Ertugay, Ümit Kahraman, Emrah Oğuz, Osman Nuri Tuncer.

**Data curation:** Serkan Ertugay, Ümit Kahraman, Emrah Oğuz, Nüzhet Seden Kocabaş.

**Formal analysis:** Serkan Ertugay, Ümit Kahraman, Esin Öztürk, Nüzhet Seden Kocabaş.

**Funding acquisition:** Serkan Ertugay, Ümit Kahraman.

**Investigation:** Serkan Ertugay, Ümit Kahraman, Emre Demir, Nüzhet Seden Kocabaş.

**Methodology:** Serkan Ertugay, Ümit Kahraman, Emre Demir, Pelin Öztürk.

**Project administration:** Serkan Ertugay, Ümit Kahraman, Mustafa Özbaran.

**Resources:** Serkan Ertugay, Osman Nuri Tuncer.

**Software:** Serkan Ertugay, Ümit Kahraman, Osman Nuri Tuncer.

**Supervision:** Serkan Ertugay, Ümit Kahraman.

**Validation:** Serkan Ertugay, Ümit Kahraman, Osman Nuri Tuncer.

**Visualization:** Serkan Ertugay.

**Writing – original draft:** Serkan Ertugay, Ümit Kahraman, Esin Öztürk.

**Writing – review & editing:** Serkan Ertugay, Ümit Kahraman, Esin Öztürk.
